# Effect of ProTaper Ultimate and ProTaper Gold on Postoperative Pain in Mandibular First Molars With Symptomatic Irreversible Pulpitis and Symptomatic Apical Periodontitis: A Randomized Control Clinical Trial

**DOI:** 10.1155/prm/9718875

**Published:** 2025-10-30

**Authors:** M. Hajwel, R. Elemam, T. Elsewify, B. Eid

**Affiliations:** ^1^Restorative Dental Sciences Department, College of Dentistry, Gulf Medical University, Ajman, UAE; ^2^Endodontic Department, Faculty of Dentistry, Ain Shams University, Cairo, Egypt

**Keywords:** postoperative pain, ProTaper gold, ProTaper ultimate, symptomatic apical periodontitis, symptomatic irreversible pulpitis

## Abstract

**Objective:**

To compare and evaluate the effect of ProTaper Ultimate root canal instrumentation on postoperative pain level of mandibular first molars showing symptomatic irreversible pulpitis and symptomatic apical periodontitis.

**Methodology:**

Forty patients referred to root canal treatment of the mandibular first molar were randomly divided into two equal groups according to the rotary file system used in the chemomechanical preparation: ProTaper Ultimate (*n* = 20) and ProTaper Gold (*n* = 20). After evaluating the preoperative pain score using the visual analog scale, the root canal treatment was performed in a single visit using a standardized protocol by a single operator. Postoperatively, the pain scores were recorded at 24-, 72-h, and 7-day intervals. Statistical analysis was performed at a significance of 0.05.

**Results:**

No significant difference was shown between the ProTaper Ultimate and the ProTaper Gold groups in the intensity of postoperative pain at all time intervals. The postoperative pain score was zero at 7 days postoperatively for both groups.

**Conclusion:**

The ProTaper Ultimate files' effect on postoperative pain is equivalent to that of the ProTaper Gold files.

**Clinical Relevance:**

The ProTaper Ultimate files' effect on postoperative pain is equivalent to that of the ProTaper Gold files. ProTaper Ultimate and ProTaper Gold rotary file systems can be equally and safely used in single-visit root canal treatment.

**Trial Registration:**

ClinicalTrials.gov identifier: NCT05747183

## 1. Introduction

Postoperative pain is an undesirable and inevitable consequence following conventional root canal treatment in many cases [1]. Chemomechanical preparation is one of the phases of root canal treatment, which might cause postoperative pain due to overinstrumentation, apical debris extrusion, and excessive enlargement of the apical foramen [2–4].

Postoperative pain is more severe and more prevalent in mandibular molars. The preoperative pulp and periapical states greatly affect the possibility and severity of the postoperative pain, being more severe in symptomatic irreversible pulpitis with symptomatic apical periodontitis. This is due to the heightened inflammatory state and the anatomical challenges of the mandibular molar teeth. The dense innervation of the mandibular molars, combined with the firing of the proinflammatory mediators associated with symptomatic irreversible pulpitis, can lead to an increased perception of pain during and after root canal therapy. Additionally, symptomatic apical periodontitis often involves periapical tissue irritation and possible infection, further exacerbating postoperative discomfort. Instrumentation in these cases must be performed cautiously, as aggressive shaping or inadequate irrigation can exacerbate inflammation by increasing the apical extrusion of debris or bacteria [5].

The design and type of instruments used during root canal cleaning and shaping influence postoperative pain. Contemporary endodontic instruments, particularly nickel–titanium files, are designed to enhance cutting efficiency while minimizing dentinal stress and apical extrusion of debris, which are critical factors associated with postoperative pain. File designs with enhanced flexibility and reduced or regressive taper can adapt better to the root canal anatomy, minimizing unnecessary dentin removal and tissue trauma [6].

The ProTaper Ultimate file system represents a significant advancement in root canal instrumentation, particularly in minimizing postoperative pain. This system combines innovative metallurgy, variable tapers, and enhanced flexibility to achieve efficient shaping while preserving dentinal structure and reducing apical stress. Its heat-treated NiTi alloy improves file flexibility, enabling safer navigation through complex canal anatomies with minimal risk of canal transportation or iatrogenic errors. The unique S-shape cross-sectional design promotes efficient debris removal while minimizing apical extrusion, a critical factor associated with postoperative pain. Additionally, the progressive taper design of ProTaper Ultimate facilitates balanced dentin removal, reducing the risk of weakening the root structure or causing unnecessary tissue irritation. The ProTaper Ultimate rotary file system exhibits a distinct progressive taper, remaining constant at the apical segment, between D1 and D3, and then progressively diminishing thereafter till D16. This configuration is reported to facilitate conservative shaping of the canal while minimizing debris extrusion [7–9].

The ProTaper Gold file system has been widely recognized for its minimal postoperative pain through its innovative design and enhanced metallurgical properties. Constructed from a proprietary gold heat-treated nickel–titanium alloy, these files exhibit superior flexibility and fatigue resistance, allowing them to adapt seamlessly to complex canal curvatures with minimal risk of transportation or procedural errors. The system's progressive taper design ensures efficient cutting and shaping while maintaining dentin integrity, reducing the potential for unnecessary canal enlargement and subsequent tissue irritation. Furthermore, its efficient debris removal capabilities minimize apical extrusion, a key contributor to postoperative discomfort. These attributes collectively make ProTaper Gold a valuable tool in achieving effective root canal preparation with reduced postoperative complications [10, 11].

Alhayki et al. [9] and Eskibağlar et al. [12] evaluated the effect of the ProTaper Ultimate on apical debris extrusion during root canal preparation of mandibular molars and premolars. Al Omari et al. [13] evaluated the impact of heating the irrigating solution on the extrusion of debris by the same files. Additionally, two more studies by Greco et al. and Waheed and Shater [7, 8], evaluated the shaping ability of this recently launched file system. So, the effect of root canal preparation using ProTaper Ultimate on postoperative endodontic pain was investigated and compared to ProTaper Gold in the current study. The null hypothesis assumes that there is no difference in the intensity of postoperative pain between PTUT and PTG files on the postoperative pain intensity following the instrumentation of mandibular first molar teeth.

## 2. Methodology

### 2.1. Study Design and Trial Registration

**Figure 1:**
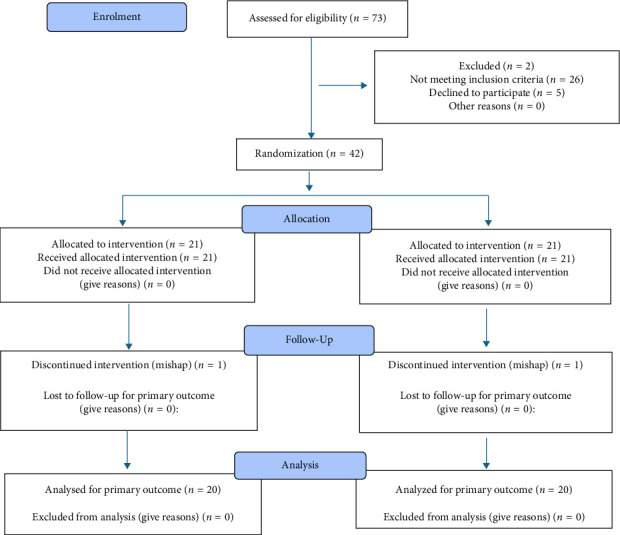
Flow diagram for the randomized control clinical trial design.

A prospective, parallel-sided, double-blinded design was followed. After obtaining ethical approval from Gulf Medical University's Institutional Review Board (IRB/COD/STD/33/June-2022), patient recruitment started on September 12th, 2022, and continued until July 1st, 2024. The study adhered to the CONSORT 2025 guidelines for reporting RCTs in endodontics, as clearly explained in Figure 1, and in accordance with the Declaration of Helsinki.

### 2.2. Patient Recruitment and Eligibility Criteria

Outpatients referred to the postgraduate clinics for root canal treatment of the mandibular first molar, fulfilling the eligibility criteria, were enrolled in the study. The enrolled patients signed an informed consent explaining the whole procedure, risks, and benefits, as well as consent for publication of data and images.

#### 2.2.1. Inclusion Criteria

ASA Class I compliant patients of both genders with canal curvature less than 25°, as calculated by Schneider's method, with pulpal diagnosis of symptomatic irreversible pulpitis and apical diagnosis of symptomatic apical periodontitis were enrolled.

#### 2.2.2. Exclusion Criteria

Pregnant patients and patients on any medication were excluded from the current study, in addition to molars showing any signs of resorption, calcification, or procedural mishaps. Immature roots, as well as mesial root canals' initial size larger than size 20 and distal canals larger than size 30, were also excluded.

### 2.3. Randomization and Allocation

All enrolled patients were assigned numbers that were randomized using the https://www.Random.org application. The first author did all the root canal treatment procedures. The third author was responsible for pain score evaluation either pre- or postoperatively. The third author was totally blind to the grouping.

### 2.4. Sample Size Calculation

Power analysis was performed based on the results obtained previously by Çanakçi et al. [14], by adopting alpha (α) and beta (β) levels of (0.05) (i.e., power = 80%) and an effect size of (2.30), yielding an estimated sample size of 38 patients. The sample size was increased to 42 patients (i.e., 21 patients per group) to account for possible dropouts during the follow-up intervals. Seventy-three patients were assessed for eligibility, and 42 patients were enrolled in the current study.

### 2.5. Treatment Protocol

Following a standardized protocol, all enrolled patients were diagnosed after collecting the patients' history and a thorough clinical examination. Pulp state was confirmed clinically after access cavity preparation in addition to thermal and electrical pulp testing before starting the procedure. Periapical state was determined based on the patients' signs and symptoms, clinical testing, and preoperative radiographic appearance. Restorative and periodontal evaluations were also performed. The third author evaluated the preoperative pain level using the visual analog pain scale (VAS). The patients indicated their pain score after a detailed explanation of the scale by the third author. The VAS is represented by a horizontal line of 100 mm with one end indicating no pain and the other indicating the worst pain (score 10) [15, 16].

One cartridge of lidocaine 2% with 1:80,000 epinephrine was administered for an inferior alveolar nerve block. After confirmation of profound anesthesia, rubber-dam isolation was applied. Access cavity was prepared using a round bur and a tapered diamond-coated stone mounted in a high-speed handpiece with copious air–water coolant. A conventional access cavity design was followed, and total deroofing of the pulp chamber was performed.

After localization and negotiation of all root canals, a size 10-K-file was used to determine the working length electronically using Root ZX II apex locator (J. Morita, Tokyo, Japan) and radiographically using intraoral periapical radiographs. A glide path was created for all canals using hand instrumentation till K-file #15. All the procedures were done under 8 × magnification using a dental operating microscope (Global Surgical Corporation, Saint Louis, USA).

For the ProTaper Ultimate group, the Slider, the Shaper, and the Finishers were used. The mesial canals were prepared until F2 (25/.08 v) and F3 (30/.09 v) for the distal canals. The procedure was done under the recommended speed and torque, 400 rpm and 4 N/cm^2^, in a brushing motion using Endo MateTC2 endodontic motor (NSK, Tokyo, Japan).

For the ProTaper Gold group, the PTG instrumentation SX (19/.04 v), S1(18/.02 v), S2(20/.04 v), F1 (20/.07 v), F2 (25/.08 v), and F3 (30/.09 v) were used. The files were used in a brushing motion under the recommended speed and torque, 300 rpm and 4 N/cm^2^, using the Endo MateTC2 endodontic motor.

The same irrigation protocol was followed in both groups tested, using sodium hypochlorite 3%, 10 mL after each rotary file over 2 min using a side-vented irrigation needle 2 mm short of the determined working length. Activation of irrigation was achieved using Ultra-X silver tip (Eighteeth Medical Technology, Changzhou, China) for 1 min in each canal. A final flush using 17% ethylenediaminetetraacetic acid was applied after washing the sodium hypochlorite using saline solution.

**Figure 2:**
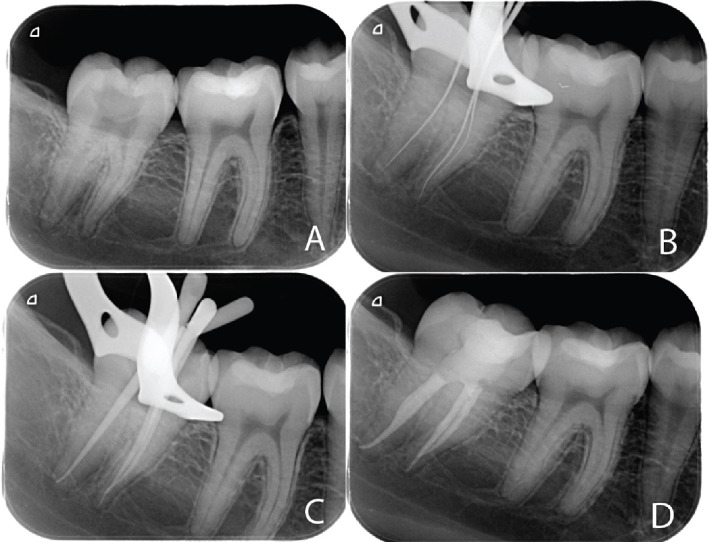
Intraoral periapical radiographs of a representative case from the ProTaper Ultimate group: (A) preoperative; (B) working length; (C) master cone; (D) postoperative.

**Figure 3:**
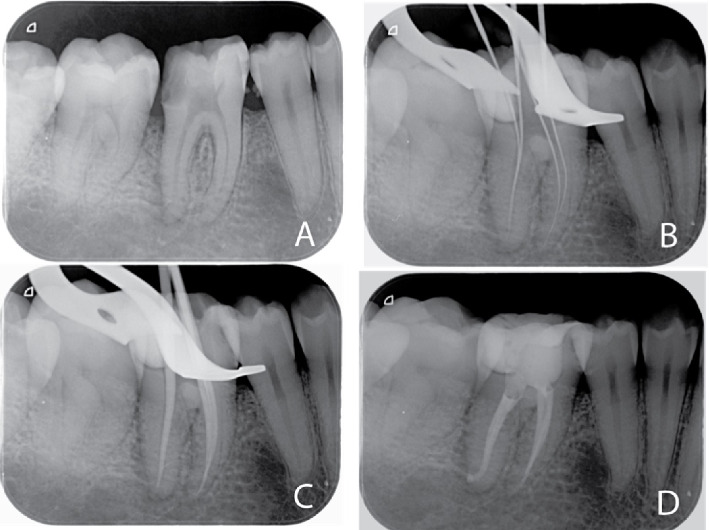
Intraoral periapical radiographs of a representative case from the ProTaper gold group: (A) preoperative; (B) working length; (C) master cone; (D) postoperative.

Single-cone obturation technique was applied using a matching master gutta-percha cone and Totalfill BC Sealer after radiographic verification of the master cone fitting. The excess was burnt using B-fill heat carrier (VDW, Germany). A postoperative intraoral periapical radiograph was acquired, as shown in Figures 2 and 3.

A final resin composite was placed to restore the coronal tooth structure till the definitive final restoration was placed. Occlusion was checked using an articulating paper, and occlusal adjustments were performed using a diamond-coated polishing bur.

### 2.6. Postoperative Pain Assessment

Postoperative pain score was recorded at 24-, 72-h, and 7-day intervals, similar to previously explained during preoperative pain evaluation. No postoperative analgesics have been prescribed in order to rule out the effect of any confounding factor. All the patients were instructed to contact the outcome assessor in case of need for intake of analgesics to be excluded from the study.

### 2.7. Statistical Analysis

R statistical analysis software (R Development Core Team, University of Auckland, New Zealand) was used to amylase the data at a significance level of 0.05. Fisher's exact test was used for the categorical data. The independent *t*-test was used for the age data as they showed a normal distribution using the Shapiro–Wilk test. Pain scores failed to demonstrate normal distribution using the Shapiro–Wilk test, and therefore, the Mann–Whitney U test and Friedman's test, followed by Nemenyi post hoc tests, were used. *p* values were adjusted for multiple comparisons using the false discovery rate (FDR) method.

## 3. Results

### 3.1. Demographic Data

In ProTaper Ultimate, there were 15 males and 5 females with a mean age of (32.40 ± 10.39) years. There were 11 males and 9 females in the ProTaper Gold group with a mean age of (34.00 ± 7.85) years. There was no significant difference between the tested groups regarding gender distribution (*p*=0.320), age (*p*=0.586), and preoperative pain score (*p*=0.847), as seen in Table 1.

### 3.2. Effect of the File System on Postoperative Pain

Regardless of measurement time, there was no statistically significant difference in the pain scores measured at different intervals between the ProTaper Ultimate and the ProTaper Gold groups (*p* > 0.05). Intergroup comparisons, median, and IQR, in addition to mean and standard deviation (SD) values for pain, are presented in Table 2.

### 3.3. Effect of Time on Postoperative Pain

**Figure 4:**
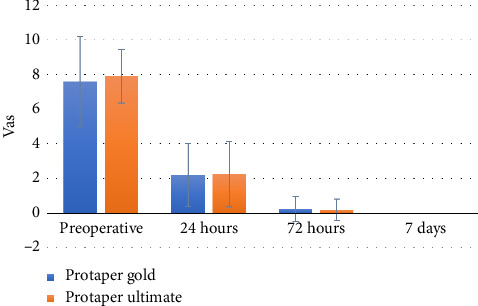
Bar chart showing pain scores' mean and standard deviation values.

Within all groups, there was a statistically significant reduction in pain scores over time (*p* < 0.001). Post hoc pairwise comparisons showed values measured preoperatively to be significantly higher than those measured at other intervals (*p* < 0.001). In addition, they showed 24-h values to be significantly higher than values measured at later intervals (*p* < 0.001). Both groups reached zero pain at the 1-week interval, as shown in Figure 4.

## 4. Discussion

Postoperative endodontic pain remains a significant clinical concern, as it directly impacts patient satisfaction and treatment outcomes. Understanding its causes is critical for devising effective pain management strategies and enhancing the overall endodontic experience. Postoperative pain is multifactorial, influenced by factors such as the extent of preoperative inflammation, canal instrumentation techniques, irrigation protocols, and the choice of obturation materials. Additionally, individual patient factors, including pain threshold and systemic conditions, play a role [3]. Postoperative pain resulting from acute periapical inflammation can be influenced by various factors, including chemical, mechanical, and microbial insults to the periapical area during root canal treatment [17]. Technical factors within the operator's control, including instrumentation, irrigation, and obturation protocols, are critical contributors. To mitigate postoperative pain, it is essential to minimize the extrusion of apical debris during the chemomechanical preparation of the root canal system.

Despite the great development in the field of rotary NiTi files, incorporating different design features, heat treatments, and alloys, all instrumentation techniques, whether manual or rotary, inevitably result in the unintentional extrusion of debris into the periapical area [18]. However, different instruments and techniques have been shown to vary in the amount of debris extrusion, with some producing less debris extrusion and consequently reducing postoperative pain [19].

The first mandibular molars were chosen for this study due to their clinical significance and anatomical complexity, which present unique challenges in endodontic treatment. These teeth are frequently encountered in dental practice and are among the most commonly treated teeth for root canal therapy. Their intricate root canal morphology, including variations in the number, curvature, and configuration of canals, makes them ideal for assessing the efficacy of different rotary instrumentation systems.

Teeth diagnosed with symptomatic irreversible pulpitis and symptomatic apical periodontitis were selected to study pain management in cases with significant preoperative inflammation and microbial load. These conditions are often associated with heightened pain perception and more pronounced postoperative responses, providing a robust framework for evaluating the effectiveness of different treatment protocols [20, 21]. Additionally, these diagnoses represent common clinical scenarios where endodontic intervention is necessary, allowing the findings to be directly applicable to routine dental practice.

Patients with systemic diseases or those taking analgesics were excluded to eliminate potential confounding factors that could influence pain perception and inflammatory responses. Systemic conditions, such as diabetes or immune disorders, may alter healing dynamics and inflammatory processes, leading to variability in postoperative outcomes. Similarly, the use of analgesics can mask pain levels, making it difficult to accurately assess the true intensity of postoperative discomfort [22]. By excluding such patients, the study ensures a more homogeneous sample, enabling a clearer evaluation of the treatment protocols' impact on postoperative pain and minimizing bias in the findings.

A single-visit root canal treatment approach was chosen to standardize the treatment protocol and minimize variability in patient outcomes. This method eliminates the potential for interappointment contamination and reduces the overall treatment time, which is often more convenient for patients [23, 24].

In the current study, a total of 73 patients were diagnosed and evaluated; however, only 47 participants were enrolled, as the rest of the patients were not eligible. Five patients refused to participate in the study. A total of 42 patients were randomized. Two patients were excluded due to intraoperative complications. One patient had an issue with the length of the procedure, and treatment was not completed in a single visit. The other patient experienced a separated instrument in the mesiobuccal canal. Therefore, only forty patients were included in the results and statistical analysis.

The VAS was selected for its simplicity, reliability, and sensitivity in assessing pain intensity. This scale allows patients to quantify their subjective pain experience on a continuous spectrum, providing detailed and nuanced data. Its ease of use and widespread acceptance in clinical research make it an ideal tool for evaluating postoperative pain, ensuring consistency and comparability with other studies in the literature [25].

No statistically significant difference between the preoperative pain scores helped obtain a fair comparison between the two tested files, as both groups shared an equivalent baseline for the pain score.

The null hypothesis tested in the current study was accepted, as no statistically significant difference in the pain score was noted between the ProTaper Ultimate group and the ProTaper Gold group at all time intervals. This can be attributed to the similarity in many manufacturing aspects such as active cutting blades without radial lands, similar surface finishing, and nearly equal nickel/titanium ratios. The ProTaper Ultimate system has several features similar to the ProTaper Gold system, including the variable taper.

Although these two file systems have not been previously compared in a randomized controlled trial regarding the postoperative pain scores, our results are in agreement with Unnikrishnan et al. [26] and Çanakçi et al. [14] in their randomized control trials. Unnikrishnan et al. [26] showed no significant difference in the postoperative pain between PTG, HEDM, and V Taper 2H. Çanakçi et al. [14] in a randomized clinical trial showed also no significant difference between the PTG and HEDM and two reciprocating REC Blue and WOG NiTi systems in postoperative pain.

As apical extrusion of debris is considered one of the major causes of postoperative pain, the results of the current study are in agreement with Eskibağlar et al. [12] in their ex vivo study evaluating the amount of apically extruded debris. Eskibağlar et al. [12] showed no statistically significant difference between the ProTaper Ultimate, TruNatomy, and Rotate file systems. Additionally, Greco et al. [8] evaluated the shaping ability of the recently launched ProTaper Ultimate compared to the BlueShaper using microCT and showed no significant differences between both files as well.

In contrast, Sharawy and Shater [7] showed that the ProTaper Gold showed less deviation than the ProTaper Ultimate at the beginning of the apical curvature. This contradiction could be attributed to the difference in the methodology adopted. Sharawy and Shater [7] evaluated both files using S-shaped canals in resin blocks, which affected their results.

Also, Alhayki et al. [9] showed that the ProTaper Ultimate extruded apical debris less than the ProTaper Gold in their ex vivo study. They attributed this to the difference in the design features between the two files, mainly due to the change in the cross section from a parallelogram to a rhomboid or S-shaped cross section in the apical 3 mm, which offers a better way for debris extrusion. This clearly explains the multifactorial nature of postoperative pain and that it cannot be solely attributed to apical debris extrusion.

Moreover, Kapoor et al. [27] in a randomized clinical trial showed that the ProTaper Gold caused significantly more postoperative pain than XPES and 2Shape. On the other hand, Ikhar et al. [28] showed that ProTaper Gold caused less postoperative pain than HEDM after single-visit root canal treatment.

Evaluating the effect of time, our results fully agree with Unnikrishnan et al. [26], Kapoor et al. [27], Ikhar et al. [28], and Çanakçi et al. [14] who showed a decreased pain score with time. A zero-pain score was reported postoperatively after 1 week. This can be explained on a molecular basis. Emad et al. [29] have shown decreased proinflammatory Interleukin 1 gene expression in addition to an increase in the gene expression of some anti-inflammatory cytokines such as Interleukin 10.

This study's findings relied on patients' self-reported pain scores using the VAS, which makes pain assessment inherently subjective. Variability in individual pain perception and interpretation may have influenced the reliability of the results. Moreover, postoperative pain can be affected by several patient- and procedure-related factors, such as individual pain thresholds, preoperative anxiety, inflammatory status, and differences in canal anatomy or operator-related variables, many of which could not be fully standardized or controlled in this study.

Further studies are deemed of value to investigate different properties of the recently launched ProTaper Ultimate file system in addition to more randomized clinical trials with larger sample sizes evaluating its effect on postoperative endodontic pain following the treatment of different pulp and periapical states.

## 5. Conclusion

In conclusion, in terms of postoperative pain, ProTaper Ultimate and ProTaper Gold rotary file systems can be equally and safely used in single-visit root canal treatment.

### 5.1. Highlight Key Points

• The ProTaper Ultimate files' effect on postoperative pain is equivalent to that of the ProTaper Gold files.
• Regardless of the rotary file tested, the postoperative pain score was zero at 7 days postoperatively.
• ProTaper Ultimate and ProTaper Gold rotary file systems can be equally and safely used in single-visit root canal treatment.

## Figures and Tables

**Table 1:** Intergroup comparisons and summary statistics for demographic data.

Parameter		PTUi	PTG	Test statistic	*p* value
Gender (*n* [%])	Male	15 (75.00%)	11 (55.00%)	1.76	0.320ns
	Female	5 (25.00%)	9 (45.00%)		

Age (Mean ± SD) (years)		32.40 ± 10.39	34.00 ± 7.85	0.55	0.586ns

Preoperative pain score	Mean ± SD	7.90 ± 1.55	7.60 ± 2.60	192.50	0.847ns
	Median (IQR)	8.00 (2.00)	8.50 (3.25)		

*Note:* ns; nonsignificant (*p* > 0.05).

Abbreviation: NA, not applicable.

**Table 2:** Intergroup, intragroup comparisons, and summary statistics for pain score.

Time	Measurements	Pain score (VAS)		Test statistic	*p* value
		ProTaper Gold	ProTaper Ultimate		
Preoperative	Mean ± SD	7.60 ± 2.60^A^	7.90 ± 1.55^A^	192.50	0.847ns
	Median (IQR)	8.50 (3.25)^A^	8.00 (2.00)^A^		

24 h	Mean ± SD	2.20 ± 1.82^B^	2.25 ± 1.89^B^	207.00	0.857ns
	Median (IQR)	2.00 (3.25)^B^	2.50 (4.00)^B^		

72 h	Mean ± SD	0.25 ± 0.72^C^	0.20 ± 0.62^C^	209.00	0.689ns
	Median (IQR)	0.00 (0.00)^C^	0.00 (0.00)^C^		

7 days	Mean ± SD	0.00 ± 0.00^C^	0.00 ± 0.00^C^	NA	NA
	Median (IQR)	0.00 (0.00)^C^	0.00 (0.00)^C^		

Test statistic		52.67	54.73		

*p* value		<0.001^∗^	<0.001^∗^		

*Note:* Values with different superscripts within the same vertical column are significantly different; ^∗^, significant (*p* < 0.05); ns, nonsignificant (*p* > 0.05).

## Data Availability

The data that support the findings of this study are available from the corresponding author upon reasonable request.

## Supporting Information

Supporting Information 1 Supporting Information I: CONSORT 2025 checklist for reporting randomized trials.

Supporting Information 2 Supporting Information II: CONSORT 2025 flow diagram for reporting randomized trials.

## References

[1] Berman L., Hk R. I. (2021). *Cohen’s Pathways of the Pulp*.

[2] Alamassi B. Y. (2017). Endodontic Postoperative Pain: Etiology and Related Factors-An Update. *International Journal of Dental Sciences and Research*.

[3] Shibu T. M. (2015). Post Operative Pain in Endodontics: A Systemic Review. *Journal of Dentistry and Oral Hygiene*.

[4] AlRahabi M. K. (2017). Predictors, Prevention, and Management of Postoperative Pain Associated With Nonsurgical Root Canal Treatment: A Systematic Review. *Journal of Taibah University Medical Sciences*.

[5] Nixdorf D. R., Moana-Filho E. J., Law A. S., McGuire L. A., Hodges J. S., John M. T. (2010). Frequency of Persistent Tooth Pain After Root Canal Therapy: A Systematic Review and Meta-Analysis. *Journal of Endodontics*.

[6] Almheiri A. R. S. A., Elsewify T., Eid B. (2023). Influence of Traverse and Waveone Gold Glider Glide Path Files on the Amount of Apically Extruded Debris. *Brazilian Dental Science*.

[7] Sharawy W. W., Shater W. H. E. (2023). Shaping Ability of Protaper Gold and Protaper Ultimate in Simulated Root Canals: A Comparative Study. *Scholars Journal of Dental Sciences*.

[8] Greco K., Iacono F., Montagna F. (2024). Shaping Ability of Protaper Ultimate and BlueShaper in Mandibular Molars: A Micro-CT Evaluation. *Front Materials*.

[9] Alhayki M. M., Eid B., Elemam R., Elsewify T. (2025). Evaluation of Apically Extruded Debris During Root Canal Preparation Using ProTaper Ultimate and ProTaper Gold: an Ex Vivo Study. *European Endodontic Journal*.

[10] Miguéns-Vila R., Martín-Biedma B., De-Deus G., Belladonna F. G., Peña-López A., Castelo-Baz P. (2021). Micro–Computed Tomographic Evaluation of Dentinal Microcracks After Preparation of Curved Root Canals With Protaper Gold, WaveOne Gold, and ProTaper next Instruments. *Journal of Endodontics*.

[11] Gagliardi J., Versiani M. A., De Sousa-Neto M. D., Plazas-Garzon A., Basrani B. (2015). Evaluation of the Shaping Characteristics of ProTaper Gold, ProTaper NEXT, and ProTaper Universal in Curved Canals. *Journal of Endodontics*.

[12] Eskibağlar M., Yeniçeri Özata M., Timis L. L. (2023). Comparison of ProTaper Ultimate, TruNatomy, and Rotate Rotary Files in Apical Debris Extrusion. *International Dental Research*.

[13] Al Omari T., Atmeh A. R., Algahtani F. N., Dkmak A., Albanna R. H. I., Tabnjh A. (2024). The Effect of Irrigation Solution Temperature and Novel Heat-Treated Rotary Files on Apical Debris Extrusion and Canal Preparation Time. *Australian Endodontic Journal*.

[14] Çanakçi B. C., Er Ö., Genç Şen Ö., Süt N. (2021). The Effect of Two Rotary and Two Reciprocating NiTi Systems on Postoperative Pain After Root Canal Retreatment on Single-Rooted Incisor Teeth: A Randomized Controlled Trial. *International Endodontic Journal*.

[15] Almasoud L., Elsewify T., Elemam R., Eid B., Dent E. J. (2025). Effect of Cryotherapy and Occlusal Reduction on Postoperative Endodontic Pain in Mandibular First Molars With Symptomatic Apical Periodontitis: A Prospective, Parallel, Double-Blinded Randomized Controlled Trial. *European Journal of Dermatology*.

[16] Shalabi M., Mahran A. H., Elsewify T. (2025). Effect of Submucosal Cryotherapy on Postoperative Pain in Maxillary Premolars With Symptomatic Irreversible Pulpitis: A Prospective, Parallel, Triple-Blinded Randomized Controlled Trial. *European Journal of Dermatology*.

[17] Wang C., Xu P., Ren L., Dong G., Ye L. (2010). Comparison of Post-Obturation Pain Experience Following One-Visit and Two-Visit Root Canal Treatment on Teeth With Vital Pulps: A Randomized Controlled Trial. *International Endodontic Journal*.

[18] Silva E. J. N. L., Carapiá M. F., Lopes R. M. (2016). Comparison of Apically Extruded Debris After Large Apical Preparations by Full-Sequence Rotary and Single-File Reciprocating Systems. *International Endodontic Journal*.

[19] Topçuoğlu H. S., Zan R., Akpek F. (2016). Apically Extruded Debris During Root Canal Preparation Using Vortex Blue, K3XF, Protaper Next and Reciproc Instruments. *International Endodontic Journal*.

[20] Gotler M., Bar-Gil B., Ashkenazi M. (2012). Postoperative Pain After Root Canal Treatment: A Prospective Cohort Study. *International Journal of Dentistry*.

[21] Segura-Egea J. J., Cisneros-Cabello R., Llamas-Carreras J. M., Velasco-Ortega E. (2009). Pain Associated With Root Canal Treatment. *International Endodontic Journal*.

[22] Segura-Egea J. J., Cabanillas-Balsera D., Martín-González J., Cintra L. T. A. (2023). Impact of Systemic Health on Treatment Outcomes in Endodontics. *International Endodontic Journal*.

[23] Nagpal A., Srivastava P. K., Setya G., Chaudhary A., Dhanker K., Dhanker K. (2017). Assessment of Coronal Leakage of Temporary Restorations in Root Canal-Treated Teeth: An In Vitro Study. *The Journal of Contemporary Dental Practice*.

[24] Shanmugam S., PradeepKumar A. R., Abbott P. V. (2020). Coronal Bacterial Penetration After 7 Days in Class II Endodontic Access Cavities Restored With Two Temporary Restorations: A Randomised Clinical Trial. *Australian Endodontic Journal*.

[25] Collins S. L., Moore A. R., Mcquay H. J. (1997). The Visual Analogue Pain Intensity Scale: What is Moderate Pain in Millimetres?. *Pain*.

[26] Unnikrishnan P., Mandke L., Padhye L. (2023). Comparison of Postoperative Pain in Single Visit Endodontics Using Heat-Treated Nickel−Titanium File Systems–A Randomized Clinical Trial. *Endodontology*.

[27] Kapoor K., Grewal M. S., Arya A., Grewal S., Prasad Shetty K. (2023). Incidence of Postoperative Pain After Single Visit Root Canal Treatment Using XP-endo Shaper, 2Shape and Protaper Gold Rotary Systems: A Prospective Randomized Clinical Trial. *European Endodontic Journal*.

[28] Ikhar A., Jaiswal A., Chandak M., Chaudhari P. (2023). Comparative Evaluation of Postoperative Pain After Single-Visit Endodontic Treatment Using Protaper Gold and Hyflex Electrical Discharge Machining Rotary File System. *Journal of Datta Meghe Institute of Medical Sciences University*.

[29] Emad A., Abdelsalam N., Fayyad D. M. (2021). Influence of Intracanal Cryotherapy on Postendodontic Pain and Interleukin-6 Expression Using Different Irrigation Protocols: A Randomized Clinical Trial. *Saudi Endodontic Journal*.

