# Supplementary material for: Effect of ProTaper Ultimate and ProTaper Gold on Postoperative Pain in Mandibular First Molars With Symptomatic Irreversible Pulpitis and Symptomatic Apical Periodontitis: A Randomized Control Clinical Trial
Source: Pain Res Manag. 2025 Oct 30;2025:9718875. doi: 10.1155/prm/9718875 (PMC12591807; doi:10.1155/prm/9718875)
Supplement: Supporting Information 2 — Supporting Information II: CONSORT 2025 flow diagram for reporting randomized trials. [file 9718875.f2.docx]

Excluded (n=2 )

Not meeting inclusion criteria (n=26)

Declined to participate (n=5)

Other reasons (n=0)

Analysis

Analysed for primary outcome (n=20)

Excluded from analysis (give reasons) (n=0)

Discontinued intervention (mishap) (n=1)

Lost to follow-up for primary outcome (give reasons) (n=0):

Discontinued intervention (mishap) (n=1)

Lost to follow-up for primary outcome (give reasons) (n=0):

Randomised (n=42)

Allocation

Follow-Up

Allocated to intervention (n=21)

Received allocated intervention (n=21)

Did not receive allocated intervention (give reasons) (n=0)

Allocated to intervention (n=21)

Received allocated intervention (n=21)

Did not receive allocated intervention (give reasons) (n=0)

Enrolment

Assessed for eligibility (n=73 )

Analysed for primary outcome (n=20)

Excluded from analysis (give reasons) (n=0)
